# Phonic and Motor Stereotypies in Autism Spectrum Disorder: Video Analysis and Neurological Characterization

**DOI:** 10.3390/brainsci11040431

**Published:** 2021-03-28

**Authors:** Evamaria Lanzarini, Jacopo Pruccoli, Irene Grimandi, Chiara Spadoni, Marida Angotti, Veronica Pignataro, Leonardo Sacrato, Emilio Franzoni, Antonia Parmeggiani

**Affiliations:** 1 Child Neurology and Psychiatry Unit, Infermi Hospital, AUSL Romagna, 47923 Rimini, Italy; evamaria.lanzarini@gmail.com; 2 Child Neurology and Psychiatry Unit, IRCCS Institute of Neurological Sciences of Bologna, 40138 Bologna, Italy; jacopo.pruccoli@studio.unibo.it (J.P.); irene.grimandi@studio.unibo.it (I.G.); chiara.spadoni83@gmail.com (C.S.); maridangotti@hotmail.it (M.A.); veronica.pignataro@libero.it (V.P.); leonardo.sacrato@aosp.bo.it (L.S.); 3 Department of Medical and Surgical Sciences, University of Bologna, 40138 Bologna, Italy; emilio.franzoni@unibo.it

**Keywords:** Autism spectrum disorder, stereotypies, phonic, vocal, video session, ADOS

## Abstract

Stereotypies are among the core symptoms of Autism spectrum disorder (ASD) and can cause significant clinical impairment. At present, phonic stereotypies in ASD have been scarcely explored. This study investigates the frequency, variability, and typologies of phonic and motor stereotypies in children with ASD and their association with clinical neurological variables. We examined 35 patients by recording standardized video sessions and administering the Autism Diagnostic Observation Schedule-Second Edition (ADOS-2). Phonic stereotypies were present in 83.0% of the patients. The most prevalent subtypes were noncommunicative vocalizations (60.0%), single syllables (37.1%), and echolalic stereotypies (22.9%). Noncommunicative vocalizations were more frequent in nonverbal patients (OR = 4.629, *p* = 0.008), while echolalic stereotypies were more represented in verbal patients (OR = 0.279, *p* = 0.028). Patients with intellectual disability (ID) showed a higher number (F(1,26) = 9.406, *p* = 0.005) and variability (F(1,25) = 7.174, *p* = 0.013) of motor stereotypies, with a higher number (F(1,26) = 13.268, *p* = 0.005) and variability (F(1,26) = 9.490, *p* = 0.005) of stereotypies involving the head/trunk/shoulders category. Patients with guttural stereotypies showed a higher variability of total motor stereotypies (OR = 1.487, *p* = 0.032) and self-directed motor stereotypies (OR = 4.389, *p* = 0.042). These results, combined with a standardized video-analysis, document the frequency and variability of phonic stereotypies among children with ASD. Correlations between specific phonic stereotypies and verbal abilities should be investigated further.

## 1. Introduction

Stereotypies are a defining feature of Autism spectrum disorder; they manifest themselves as bodily movements, called motor stereotypies, or production of sounds, called phonic stereotypies [1]. Motor stereotypies are predictable and non-goal directed movement patterns, which are repeated continuously for a period in the same form and on multiple occasions and are frequently distractible. They tend to appear when the child is focused on an activity or during periods of boredom, anxiety, excitement, or fatigue [2,3,4,5]. Phonic stereotypies range from repetition of simple vocalizations to the purposeless emission of more complex sounds, such as words or phrases [6,7].

Motor stereotypies and phonic (vocal) stereotypies have been described in children with Autism spectrum disorder (ASD) and are one of the core symptoms of ASD [8], along with restricted interests and activities [9]. However, they are not pathognomonic of ASD, as they can also be observed in children with intellectual disability, sensory deprivation [10], or even in typically developing (TD) children [2]. A recent systematic review indicates the presence of motor stereotypies in 88% of patients with ASD [11]. Regarding the prevalence of phonic stereotypies, we do not have definitive data due to the variability of its definition [7].

Based on the presence or absence of other developmental problems, stereotypies are subclassified into primary (physiological) and secondary (associated with neurological or psychiatric conditions or sensory impairment) categories [12].

Regarding the specific conditions that may be present with secondary stereotypies, these symptoms are common in fragile X syndrome (FXS), with and without ASD; ASD is common in males with FXS as well. Testing for fragile X syndrome (FXS) is among the genetic, neurological, and psychiatry practice parameters for the assessment of stereotypies [13,14].

The pathophysiology underlying these repetitive behaviors remains largely unknown; reasons advanced for their manifestation range from psychological factors to neurobiological abnormalities. While psychological factors cannot be ruled out as contributing to these behaviors, it has largely been demonstrated that biological abnormalities, including neurochemical, anatomical, and genetic factors, play a significant role in their occurrence [15]. 

Regarding motor stereotypies, several studies have found a correlation between the quantity and intensity of motor stereotypies and the severity of the clinical picture. Goldman and collaborators observed that children with autism tend to show higher rates and more types of motor stereotypies than impaired children without autism. They also found a positive correlation with the severity of the clinical picture, that is, with the highest occurrence of repetitive behaviors when both autism and intellectual disability are present. This association was observed to be stronger in females [16]. Moreover, self-injurious stereotypies appear to be more common in patients with ASD compared to controls with typical development (TD). When ASD is comorbid with intellectual disability, self-injurious stereotypies are especially more frequent [17].

As for phonic stereotypies, fewer clinical descriptions are present in literature. In a study by Mayes and Calhoun, parents reported that 85% of 777 children with autism presented atypical, repetitive vocalizations or speech [18]. MacDonald and colleagues compared the duration of vocal stereotypies among TD children with and without ASD, documenting an increased number of vocal stereotypies in children with ASD and longer stereotypies more frequent in older children [19]. Van Santen and colleagues investigated repetitive speech in ASD and TD children and distinguished between self-repeats (repetitions of sounds produced by the child him/herself) and echolalia (repetition of what is uttered by an interlocutor). The authors found a higher presence of echolalia in ASD children, while no difference was reported for self-repeats between the two groups [7]. It is relevant to note that some authors have recently questioned the understanding of phonic stereotypies as mere deficits, suggesting a possible role of these symptoms in the communication patterns adopted by children with ASD [20]. 

The accurate categorization of vocal behaviors in infants and children with ASD represents a relevant clinical issue, since they may serve as early diagnostic markers of autism [21].

The literature provides little information on the relationship between motor and phonic stereotypies. A relevant study on this topic is a retrospective chart review by Gosh and colleagues, comparing motor and phonic stereotypies in TD and ASD children. Stereotypies were more frequently movement-related than voice-related in each group. Phonic stereotypies were more represented in ASD than in TD children, with 57% vs. 11% of patients presenting both phonic and motor stereotypies. Interestingly, the authors of this study did not find any patients exhibiting only phonic stereotypies. Another significant result is that none of the patients presented a combination of complex phonic/simple motor stereotypies. This study classified phonic stereotypies as “simple” and “complex” but did not provide a description for different types of phonic stereotypies [1].

Despite their clinical relevance for the identification and classification of stereotypies in patients with ASD, studies on these phenomena are rarely represented in literature. This is particularly true for phonic stereotypies [21]. Technical considerations, such as poor-quality audio or difficulties in having children with ASD to fix the video-camera, have been indicated as possible reasons [16]. 

The aim of this study is to provide a clinical description of motor and phonic stereotypies—especially the latter—in a sample of children with ASD and to investigate possible correlations between these symptoms and clinical neurological features. We suggest that a greater frequency of phonic stereotypies may correlate with a greater prevalence of motor stereotypies and with impairments in multiple clinical neurological domains. 

## 2. Materials and Methods

### 2.1. Participants

Participants were recruited among children of up to 18 years of age who were evaluated at our hospital unit between 2014 and 2017 and who received a diagnosis of ASD according to the Diagnostic and Statistical Manual of Mental Disorders, Fifth Edition (DSM-5) criteria. Parents gave informed consent to the processing of personal data at the time of the clinical evaluation; approval by the local ethical committee was also obtained (Comitato Etico Indipendente Azienda Ospedaliero-Universitaria di Bologna, Policlinico Sant’Orsola—Malpighi n°346/2017/O/OssN). The diagnosis was performed by clinicians with expertise in ASD (EL, AP), who individually examined all patients, and was backed by two trained psychologists (MA, VP) through the administration of the Autism Diagnostic Observation Schedule-Second Edition (ADOS-2). Each child was administered the ADOS-2 in the same room as the previous examinations. During the administration of the ADOS-2, videos were also recorded as part of the evaluation’s protocol normally followed in our unit. When Asperger’s syndrome was suspected, the Australian Scale for Asperger’s Syndrome (ASAS) was also administered. 

Every child underwent EEG examination, brain MRI, and genetic testing (CGH array) as part of the normal ASD assessment protocol. EEG results were classified into “positive” (asymmetry of background activity or presence of epileptiform abnormalities) and “negative” (absence of both asymmetry of background activity and epileptiform abnormalities). Brain MRI results were classified into “positive” (evidence of brain malformations or signs of previous cerebrovascular injuries) and “negative” (absence of both brain malformations and signs of previous cerebrovascular injuries). Genetic tests for FXS were not systematically performed in this sample. 

Cognitive and psychomotor levels were assessed with specific scales according to the age of the participants (The Bayley Scales of Infant and Toddler Development, 3rd edition; The Wechsler Preschool and Primary Scale of Intelligence, 3rd edition; and The Wechsler Intelligence Scale for Children, 4th edition). Twenty children (6 females, 14 males) showed various degrees of intellectual disability; 11 children (3 females, 8 males) had a normal cognitive development. In 4 cases (all males), the cognitive level was not available. Twenty-three children (4 females, 19 males) had verbal communication limited to one word or spoke no words at all. For these reasons, they were considered “nonverbal”. No participant was on medication at the time of the video recording.

### 2.2. Study Design 

The study was designed as a single-center, observational and retrospective study.

### 2.3. Procedures

The first 15 min of each archived video were considered. The quantity of phonic and motor stereotypies and the parts of the body that these involved were scored by two experienced clinicians (EL, AP). 

The examiners scored as motor stereotypy any apparently purposeless repetitive movement that they observed at least twice non-contiguously. Motor stereotypies were categorized following the classification developed by Goldman and collaborators [16]. According to this classification, the following mutually exclusive categories were considered for motor stereotypies: face (grimacing, lips, tongue movements, opening of the mouth), head/trunk/shoulders (head tilting, shaking, nodding; body rocking, bending, scrunching; arching the back; shrugging the shoulders), arm/leg (flapping, crossing the arms on the chest, stamping the feet), hand/finger (shaking, tapping, waving, clapping, opening-closing, twirling the hand or fingers), hand/finger with object (shaking, tapping, twirling an object), gait (pacing, jumping, running, skipping, spinning), self-directed (covering the ears, mouthing, smelling, rubbing the eyes, tapping the chin, banging the arms against the body, slapping oneself or an object or a surface, and touching the genitals), and lateral-gaze stereotypies (looking at objects or fingers ‘out of the corner of one’s eye’). The clinicians assigned each observed stereotypy to one of the eight types and counted the number of types for each child to assess the variability of the stereotypies.

The examiners scored as a phonic stereotypy any instance in which the child expressed an apparently purposeless sound and repeated it at least twice.

Since a validated classification system does not exist for phonic stereotypies, we reviewed existing literature [7,22,23,24,25] to extract clinical descriptions of phonic stereotypies and classify them according to their increasing complexity. As a result, we considered the following subgroups: single phonemes (e.g., “mmm”), guttural sounds, noncommunicative vocalizations, syllables, echolalic stereotypies, and complex stereotypies (e.g., complex sounds or short songs). Given the relatively small dimensions of the room where the videos were recorded and the good sound quality of the recording, we are confident that our results are not linked to the quality of the audio recording. Variability of stereotypies was assessed as the total number of qualitatively different stereotypies in a subclass of stereotypies (e.g., motor stereotypies). 

All the videos were examined independently by each of the two examiners.

### 2.4. Statistical Analysis

Descriptive analyses for demographics, clinical variables, and stereotypies were performed. Chi-square and Fisher’s exact tests were used to assess correlations between clinical variables and the presence of stereotypies. Student t-tests and Mann–Whitney tests were used to investigate possible differences concerning the number and variability of stereotypies between selected clinical subgroups. Shapiro–Wilk and Levene tests were used to assess normality of data distribution and homogeneity of variance. Based on significant correlation coefficients between clinical variables and stereotypies, logistic regressions and analyses of covariance (ANCOVA) were conducted to identify specific contributions of different factors, adjusting for sex and age. Statistical analyses were conducted using JASP, version 0.14.1 for Windows. The adopted alpha error rate was 0.05 (two-tailed), with conservative statistical power of 95%. 

## 3. Results

### 3.1. Demographics and Clinical Variables

Thirty-five patients were enrolled in this study, 26 males and 9 females, aged between 1 and 11 years (mean age 4.32 +/− 2.39 years). Twenty-eight children were affected by an idiopathic ASD (2 of them had Asperger’s syndrome), while in 7 cases, a specific clinical syndrome or a genetic mutation was present (1 Turner syndrome, 1 Down syndrome, 1 Noonan syndrome, 1 fetal alcohol syndrome, 1 Rett syndrome, 1 carried a dupXp22.2, and 1 carried a dup19p13.12). 

Demographic data and frequency measures are reported in Table 1. Female patients presented a higher frequency of cognitive impairment (CI) (*p* = 0.038). The presence of verbal language was not significantly related either to sex or to the presence or absence of CI. The inter-rater reliability Cohen’s Kappa ranged from K = 0.75 (for phonic stereotypies) to K = 0.85 (for motor stereotypies), indicating consistency and agreement between the observations and evaluations of the two clinicians who scored the videos.

### 3.2. Quantity, Variability, and Correlations of Phonic Stereotypies 

Twenty-nine children (83.0%) exhibited phonic stereotypies during the recording time. The frequency and types of phonic stereotypies are reported in Table 2. The presence and variability of phonic stereotypies considered as a broad category and of specific phonic stereotypies were not significantly correlated with the presence or absence of CI, syndromic etiology, or sex. EEG and brain MRI abnormalities were not associated with differences in phonic stereotypies manifestation. 

The presence of phonic stereotypies as a broad category and their total number and variability were not correlated with the presence of verbal language. In considering specific phonic stereotypies, noncommunicative vocalizations were more prevalent in nonverbal patients (OR-adjusted = 4.629, 95% confidence interval 0.408–2.657, *p* = 0.008), while echolalic stereotypies were more represented in verbal patients (OR-adjusted = 0.279, 95% confidence interval −2.348–−0.136, *p* = 0.028).

### 3.3. Quantity, Variability, and Correlations of Motor Stereotypies

Frequency and types of motor stereotypies are reported in Table 2. Thirty-four (97.0%) out of the 35 children considered in this study showed at least one type of motor stereotypy during video recording. 

As for motor stereotypies as a broad category, CI was significantly correlated to a higher number (F(1,26) = 9.406, *p* = 0.005) and variability (F(1,25) = 7.174, *p* = 0.013) of motor stereotypies. 

As for specific motor stereotypies, children with CI showed a higher number (F(1,26) = 13.268, *p* = 0.005) and variability (F(1,26) = 9.490, *p* = 0.005) of stereotypies involving the head/trunk/shoulders category. CI was not significantly related to the total number or variability of face, arm/leg, hand/finger, hand/finger with object, gait, self-directed, and visual stereotypies.

### 3.4. Phonic and Motor Stereotypies—Differences between Subgroups

Guttural stereotypies were significantly more frequent among children with a higher variability of total motor stereotypies (OR-adjusted = 1.487, 95% confidence interval 0.034–0.760, *p* = 0.032) and with self-directed motor stereotypies (OR-adjusted = 4.389, 95% confidence interval 0.056–2.902, *p* = 0.042). No significant differences were found between the remaining phonic and motor stereotypies. Visual/gaze stereotypies were more frequent among children with a smaller presence (OR = 0.753, 95% confidence interval 0.021–2.896, *p* = 0.047) of phonic stereotypies. 

## 4. Discussion

Our study reports on the clinical variability of phonic and motor stereotypies in a sample of children with ASD. This has been the first study to investigate different neurological characteristics between patients with and without specific phonic stereotypies and to document, with video analysis, the frequency of specific phonic stereotypies subtypes in a population of patients with ASD. Standardized video-analyses have been successfully used in the assessment and treatment of other neurodevelopmental disorders, such as Rett Syndrome, and may help recognize and classify different types of stereotypies [22]

### 4.1. Phonic Stereotypies

Scientific literature lacks a validated classification for phonic stereotypies. Nonetheless, a series of studies have provided clinical descriptions of these symptoms. We list here some of the characterizations that we found in selected works on this topic: repetitive sounds, repetitive words, repetitive phrases [23], echolalia, and self-repetitions [7]; non-verbal vocalizations or vocal stimming [24]; repetitive multiple syllable sounds, repetition of phrases, delayed echolalia [25]; tics and tic-like vocalizations, vocalizations as part of stereotypies, vocalizations as part of dystonia or chorea, continuous vocalizing behaviors such as groaning or grunting, pathological laughter and crying, and vocalizations resembling physiological reflexes [26]. We have taken clinical descriptions of phonic stereotypies from these articles and have organized them according to their increasing complexity.

Among our patients, 83.0% presented phonic stereotypies. This result is significantly greater if compared to the findings reported by one of the few studies investigating this topic [1]. Notably, the group of 28 patients with neurodevelopmental disorders considered in the study of Ghosh and collaborators revealed a total frequency of phonic stereotypies equal to 57%. However, that group included only 6/28 patients with autism and 1/28 patient with Asperger syndrome. Given the markedly mixed composition of that group, it is difficult to establish a direct comparison. 

In our sample, noncommunicative vocalizations were the most frequent type of phonic stereotypies (60.0%) and were significantly more prevalent among non-verbal patients. According to the results reported in previous research, noncommunicative vocalizations in four patients with language impairment were responsive to a specific behavioral treatment [27]. 

Echolalic stereotypies were considerably represented in our population (22.9%) and significantly more present in verbal patients. This apparently intuitive finding is in contrast with previous research showing echolalia to be almost equally present among patient with ASD with and without language impairment [7]. Concerning guttural sounds, a frequency of 20.0% was documented in our population. Notably, guttural sounds were significantly more prevalent in patients with a greater number of self-directed stereotypies and greater variability of motor stereotypies, which suggests that they may be associated with more complex clinical pictures. 

Despite the great frequency of phonic stereotypies in our sample, we could not link their comprehensive presence to any of the clinical features that we took into consideration. 

### 4.2. Motor Stereotypies

Thirty-four out of 35 patients (97.2%) displayed at least one type of motor stereotypy. The cited study of Goldman and collaborators [16] found a lower frequency of motor stereotypies among patients with low-functioning ASD (70.6%) and high-functioning ASD (63.6%). However, the authors analyzed videotapes that were recorded between 1985 and 1988 and were characterized by poor audio and reproduction of facial stereotypies. Thus, differences in the video-analyses could be partially responsible for the dissimilar frequency of stereotypies documented in their and our study.

Our study confirms the association between the severity of the clinical picture and the frequency and repertoire of repetitive motor behaviors already described in the literature [15]. In fact, we found that children with ASD and CI exhibited higher rates and a broader repertoire of repetitive movements than children with ASD without CI. When ASD is associated with CI, a greater disruption of the neurochemical transmission and of the normal neural circuit function is to be expected. This could explain the higher frequency and variability of repetitive patterns of motor behaviors observed in these children [2]. Cognitive impairment was significantly correlated with the higher number and variability of stereotypies in the head/trunk/shoulders categories (*p* = 0.005). 

Interestingly, we found that 40% of children manifested atypical staring at fingers or objects. Discording opinions are reported in literature: some describe these stereotypies as typical of children with ASD [16]; others show that this is not always the case [28]. Nonetheless, the age of the patients enrolled in the study of Baranek (9–12 months) vary considerably from the mean age in our study (4.32 years) and in the one of Goldman and colleagues (4 years, 11 months). We hypothesize that a lower frequency of staring stereotypies in the study of Baranek could partially be due to a significantly lower mean age of the sample.

We did not find a significant difference between children with or without CI in terms of self-directed stereotypies. Self-injurious behaviors are normally associated with severe intellectual disability as well as autism, and their frequency is greater among children with both ASD and CI [17,29]. In our study, no self-directed repetitive movement was self-injurious, and we believe that it is for this reason that we did not find any difference between the two subgroups.

### 4.3. Future Directions and Limitations

Our results suggest that the clinical observation of repetitive motor behaviors exhibited by children with ASD may provide clues to the severity of their clinical picture and lead to an evaluation of cognitive impairment or a secondary cause of autism. The understanding of the mechanisms that give rise to behavioral stereotypies is particularly important when they become self-injurious or socially disruptive, requiring an intervention to control and reduce them.

Even though our study presents a few limitations, such as its retrospective nature and the lack of a control population, it also builds on strong points. First, the two clinicians who performed the scoring (EL and AP) watched all the videos independently, with an acceptable inter-rater reliability. The use of videos, recorded in a clinical and standardized evaluation setting, made it possible to maximize the reproducibility of the results. Second, the use of recorded videos to describe the clinical appearance of stereotypies allowed for greater accuracy than the reliance on parental questionnaires. This is especially true when videos are recorded in standardized settings to minimize environmental biases [30]. Third, few studies so far have considered both motor and phonic stereotypies as our research does. A limitation of this study is the lack of systematic testing for FXS. Since stereotypies are frequent in FXS with and without ASD, future studies should add this test to the assessment protocol. Moreover, the lack of a validated classification for phonic stereotypies in literature represents a limitation not only for this study but also for all the works concerning stereotypies in ASD; thus, further research should verify and expand our results in wider populations.

## 5. Conclusions

Standardized video-analyses may help recognize and classify different types of stereotypies. Phonic stereotypies represent a relevant clinical expression of ASD. Valuable differences in the presentation of specific phonic stereotypies between patients with and without the development of a structured language emerged in our sample. Furthermore, patients with a comorbid CI showed a greater expression of motor stereotypies. These differences were independent of the age and sex of the patients. Additional studies are needed to investigate the relationship between presence of stereotypies and developmental status in children with ASD.

## Figures and Tables

**Table 1:** Demographic and main clinical variables of the sample.

Variables	Subtypes	Frequency
Gender	Male	26 (74.3%)
	Female	9 (25.7%)
Etiology	Idiopathic	28 (80.0%)
	Syndromic	7 (20.0%)
Cognitive impairment (CI)	Yes	11 (31.4%)
	No	20 (57.1%)
	Nonvaluable	4 (11.4%)
Language development	Verbal	12 (34.3%)
	Nonverbal	23 (65.7%)

**Table 2:** Frequency and types of phonic and motor stereotypies.

Categories of Phonic Stereotypies	Frequency	Categories of Motor Stereotypies	Frequency
Phonic—total	83.0%	Motor—total	97.2%
Single phonemes	11.4%	Face	42.9%
Noncommunicative vocalizations	60.0%	Head/trunk/shoulders	54.3%
Syllables	37.1%	Hand/finger	34.3%
Echolalic stereotypies	22.9%	Hand/finger with object	54.3%
Complex sounds	11.4%	Arm/leg	57.1%
		Gaze	37.1%

## Data Availability

The datasets used and analyzed during the current study are available from the corresponding author on reasonable request.
